# Molecular map of cGAS-STING pathway-related genes in bladder cancer: the perspective toward immune microenvironment and prognosis

**DOI:** 10.18632/aging.205442

**Published:** 2024-01-17

**Authors:** Dong Wei, Ying Liu, Ying Yuan, Yishuai Li, Fangchao Zhao, Xuebo Qin

**Affiliations:** 1 Department of Urology, Hebei General Hospital, Shijiazhuang 050000, China; 2 Department of Neurology, Xingtai Third Hospital, Xingtai 054000, China; 3 Department of Thoracic Surgery, Hebei Chest Hospital, Shijiazhuang 050000, China; 4 Department of Thoracic Surgery, The Second Hospital of Hebei Medical University, Shijiazhuang 050000, China; 5 Department of Thoracic Surgery, The Fourth Hospital of Hebei Medical University, Shijiazhuang 050000, China

**Keywords:** cGAS-STING pathway, bladder cancer, tumor immune, immune microenvironment, immune checkpoint inhibitors

## Abstract

Background: The cGAS-STING pathway emerges as a pivotal innate immune pathway with the potential to profoundly influence all facets of tumor initiation and progression. The prognostic significance and immunological role of cGAS-STING pathway-related genes (CRGs) in individuals diagnosed with bladder cancer (BLCA) have not yet been fully elucidated.

Methods: Performed unsupervised cluster analysis to identify distinct clusters. Utilizing LASSO and multivariate Cox regression analysis to construct a prognostic risk model. The IMvigor210, GSE13507 and GSE78220 cohorts were utilized to explore the potential value of risk score in immune therapy response and survival prediction.

Results: A risk model was developed utilizing four CRGs in order to forecast the overall survival (OS) of BLCA patients. The risk score to be a standalone risk factor, which was further corroborated by the external validation set obtained from the GEO database (GSE13507). We established an integrated nomogram that combined risk scoring and clinical information, exhibiting commendable clinical practicality in predicting the overall survival period of BLCA patients. It is noteworthy that risk score could differentiate tumor microenvironments among different risk groups and individuals who were more responsive to immunotherapy in IMvigor210 and GSE13507 cohorts. *In vitro* experiments, we noted an up-regulation of IRF3 and IKBKB upon the activation of the cGAS-STING pathway. Conversely, the activation of the cGAS-STING pathway resulted in a down-regulation of POLR3G and CTNNB1.

Conclusions: CRG risk model shows promise as a potential stratification approach for bladder cancer patients.

## INTRODUCTION

Bladder cancer (BLCA) is the prevailing malignant neoplasm affecting the urinary tract, arising from the mucosal lining of the bladder. It ranks ninth among the most frequently diagnosed cancers globally [1]. According to recent research findings, urothelial cell carcinomas account for a significant majority of BLCA, specifically approximately 90% [2]. Tobacco smoking, along with occupational exposure to environments containing aromatic amines, PAHs, and other chemical substances, represents the primary contributing factors to the incidence of BLCA [3]. The recurrence rates and long-term survival rates of BLCA are strongly influenced by the malignancy characteristics of the disease, specifically the degree of invasiveness. Multiple staging and grading systems have been proposed to evaluate prognoses in BLCA. The tumor node metastasis (TNM) classification system stands out as the prevailing and extensively employed method among various approaches [4]. Nevertheless, substantial evidence suggests that patients diagnosed with BLCA who share the same pathological grade or clinical stage may exhibit diverse prognoses and survival rates, which suggests that the existence of additional factors beyond those encompassed by the current TNM classification systems that exert a notable influence on patient outcomes [5]. In recent years, numerous studies have delved into the examination of genes associated with the prognosis of BLCA and have developed prognostic models for BLCA survival [6, 7].

Cyclic GMP-AMP synthase (cGAS) functions as a DNA sensor, responsible for the recognition of cytoplasmic DNA and subsequent activation of the downstream stimulator of interferon genes (STING) [8, 9]. The cGAS-STING DNA sensing pathway has been acknowledged as a crucial component in the innate immune response. The STING pathway plays a pivotal role in regulating the immune interactions within tumors and exerts diverse effects on tumor cells [10]. In recent investigations, emerging research has elucidated the intricate connection between the cGAS-STING pathway and tumor immunity. The activation of the cGAS-STING pathway in antigen-presenting cells, for instance, stimulates the synthesis of Tap2 and MHC-I molecules, which has the potential to enhance immune surveillance of tumors [11]. The activation of the cGAS-STING signaling pathway led to increased levels of type I interferons (IFNs) and tumor-infiltrating lymphocytes (TILs), consequently triggering an immunogenic reaction [12, 13]. These results indicate that cGAS-STING pathway-related genes, known as CRGs, hold promise as therapeutic targets in patients with BLCA. Furthermore, these CRGs may be linked to immune infiltration in BLCA patients.

This study integrates RNA-seq data and clinical information from the TCGA and GEO databases, culminating in the identification of four CRGs. Moreover, a novel risk assessment instrument has been formulated with the purpose of forecasting the prognostic significance of BLCA and appraising the immune response. This prognostic tool possesses the potential to forecast and enhance the therapeutic outcomes of BLCA immunotherapy and clinical management.

## MATERIALS AND METHODS

### Dataset and preprocessing

We incorporated two distinct BLCA cohorts from the TCGA and GEO databases. The retained samples adhered to the following criteria: (1) Possessed gene expression profiles and comprehensive survival information; (2) Exhibited a survival duration exceeding 30 days. Ultimately, the GSE13507 cohort encompassed 165 patients, while the TCGA-BLCA cohort comprised 395 patients. The data of the GEO database were annotated using the Illumina human-6 v2.0 expression beadchip platform. For the TCGA-BLCA cohort, the transcriptomic data in the TPM format was annotated using the GENCODE database (version GRCh38). CRGs were downloaded from the PathCards database (https://pathcards.genecards.org/), encompassing a total of 103 CRGs. In the differential analysis, the data from normal bladder samples in the GTEx database served as the control, and the “limma” package was employed for conducting the differential analysis. The threshold selection was adj.*P*<0.05, | log2 (fold change)| >1. We chose CRGs with discernible disparities for subsequent investigation. The immunohistochemical staining images of gene expression in BLCA tissue and normal bladder tissue were downloaded from the Human Protein Atlas (HPA) database.

### Consensus clustering

An unsupervised consensus clustering analysis was conducted using the “ConsensusClusterPlus” package. This analysis aimed to identify distinct clusters within the dataset based on the expression profiles of prognostic CRGs with a significance level of *P* < 0.05. The optimal number of clusters was determined by utilizing the cumulative distribution function (CDF) curve in combination with the K-means algorithm. The clustering algorithm was performed 100 times to ensure robustness, with each repetition randomly sampling 80% of the data. All features were included in the clustering process, allowing for comprehensive analysis. The Euclidean distance metric was employed to calculate the dissimilarity between data points, measuring the straight-line distance in a multi-dimensional space. The “km” option chosen for the clusterAlg parameter refers to the k-means algorithm, which partitions the data into clusters by minimizing the within-cluster sum of squares.

### Building and evaluating prognostic model

In the TCGA-BLCA cohort, we employed univariate Cox analysis to identify prognostic-related CRGs. To eliminate irrelevant genes, we utilized the least absolute shrinkage and selection operator (LASSO) method with a penalty parameter (λ). The parameter was selected using k-fold cross-validation, with k set to 10. To establish a formula for calculating risk scores, we conducted multifactorial Cox analysis and obtained correlation coefficients along with corresponding gene expression values. We ensured the assumption of proportional hazards through an assumption check. Model goodness-of-fit was assessed using the likelihood ratio test and akaike information criterion (AIC). Based on the application of this formula, patients were classified into high-risk and low-risk groups using the computed median score. To validate the model, we analyzed the GSE13507 cohort.

### Nomogram construction

A more accurate prognostic nomogram was developed by integrating clinical characteristics with a risk score, utilizing the “rms” package. The performance evaluation of the nomogram was conducted by employing calibration and ROC curves.

### Enrichment analysis

Utilized the “limma” package (FDR < 0.05, log2 (FC) > 1) to discern differentially expressed genes (DEGs) between high- and low-risk groups. The default gene set “c2.cp.kegg.v7.4.symbols.gmt” was employed, and gene set enrichment analysis (GSEA) as well as gene ontology (GO) enrichment were performed on distinct risk groups utilizing the “clusterProfiler” package. Moreover, we utilized the gene set “h.all.v7.5.1.symbols.gmt” from MSigDB and employed the software package “GSVA” to perform gene set variation analysis (GSVA). This approach allowed us to examine the heterogeneity of diverse biological processes. The enrichment analysis results were presented using the R packages “ggplot2” and “GseaVis”.

### Immune correlation analysis

The CIBERSORT algorithm and single-sample gene set enrichment analysis (ssGSEA) algorithm were utilized to assess the prevalence of immune cells in different samples. The ssGSEA algorithm assessed the activation status of immune-related functions. There are currently six recognized immune subtypes, namely wound healing, inflammatory, lymphocyte deficient, immunologically quiet, and TGF-dominant [14]. In order to evaluate these subtypes in the TCGA-BLCA samples and compare them with the constructed risk model, an analysis of the differences was conducted using the chi-square test. The correlation between high-risk and low-risk cohorts in terms of pathological staging was evaluated using the same methodologies.

### Immunotherapy dataset

From the preceding literature, two cohorts (IMvigor210 and GSE78220) that received immune checkpoint blockade (ICB) treatment were obtained for the purpose of generating a risk score. The effectiveness of their risk scores in the immunotherapy cohort was validated using the same formula. The dataset of IMvigor210 consisted of 298 patients diagnosed with advanced urothelial carcinoma who received Atezolizumab, an anti-PD-L1 medication, as part of their treatment [15]. Patients diagnosed with metastatic melanoma were subjected to treatment with pembrolizumab, an anti-PD-1 drug, as indicated in the GSE78220 dataset [16]. Firstly, these cohorts were used to explore the potential value of the risk score derived from our prognostic risk model in predicting the response to immune therapy. This analysis aimed to assess whether the risk score could differentiate tumor microenvironments among different risk groups and identify individuals who were more likely to respond to immunotherapy. Secondly, the above datasets were used to assess the generalizability and reliability of our risk model in forecasting the OS of BC patients.

Immunophenoscores (IPS) were retrieved from The Cancer Immunome Atlas (TCIA) database for BLCA [17]. To evaluate the potential efficacy of immunotherapy, we performed a comparative analysis between the high-risk and low-risk groups using Immunophenoscores (IPS) retrieved from the TCIA database. The IPS scores were compared to predict the sensitivity to immunotherapy. Additionally, we employed the Tumor Immune Dysfunction and Exclusion (TIDE) algorithm to assess the likelihood of response to immune checkpoint inhibitor (ICI) treatment. Lower TIDE scores indicate a higher probability of responding to ICI treatment with more significant effects.

### Cell lines culture

The T24 BLCA cell line was obtained from the Cell Resource Center of Shanghai Life Sciences Institute. The cells were cultured in RPMI-1640 medium (Gibco BRL, USA) supplemented with 10% fetal bovine serum (FBS) and 1% streptomycin and penicillin (Gibco, Invitrogen, USA). The cells were maintained in a controlled environment with 5% CO_2_, 95% humidity, and a temperature of 37° C.

### Reverse transcription-quantitative polymerase chain reaction (RT-qPCR)

Cell lines were subjected to total RNA extraction using TRIzol (15596018, Thermo Fisher Scientific, USA) according to the manufacturer’s protocol. Subsequently, cDNA was synthesized using the PrimeScriptT-MRT kit (R232-01, Vazyme, China) for further analysis. The mRNA expression levels were determined through real-time polymerase chain reaction (RT-PCR) using SYBR Green Master Mix (Q111-02, Vazyme). The obtained data were subsequently normalized to the expression level of GAPDH mRNA. The expression levels were determined utilizing the 2^−ΔΔCt^ method, and all primers were procured from Tsingke Biotech (Beijing, China). Different concentrations of ADU-S100 (STING agonist), namely 0μM, 10μM, 20μM, and 40μM, were introduced into the culture medium. The cells were then cultured for a duration of 24 hours, followed by the execution of RT-qPCR.

### Availability of data and material

The original contributions presented in the study are included in the article/Supplementary Material. Further inquiries can be directed to the corresponding authors.

## RESULTS

**Figure 1:**
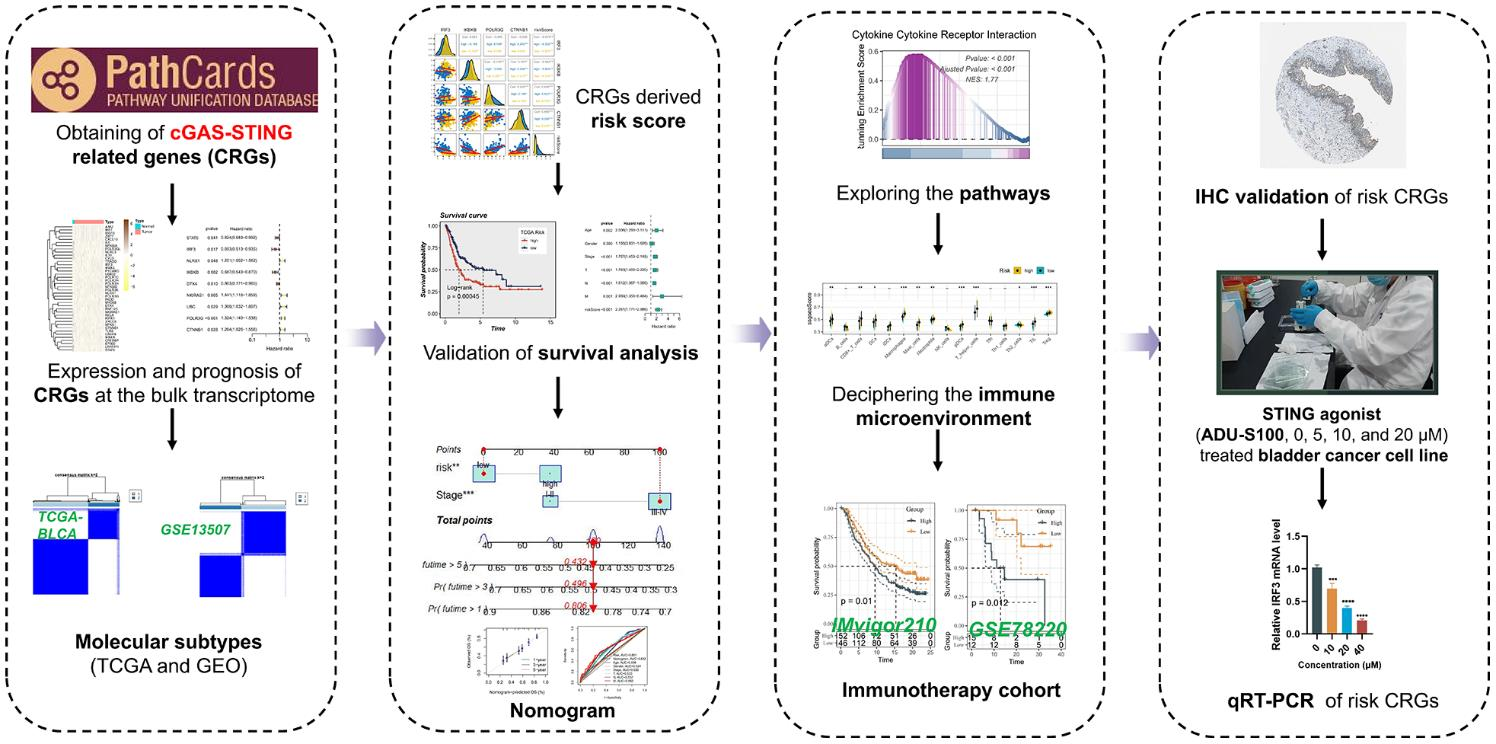
Workflow of our study.

**Figure 2:**
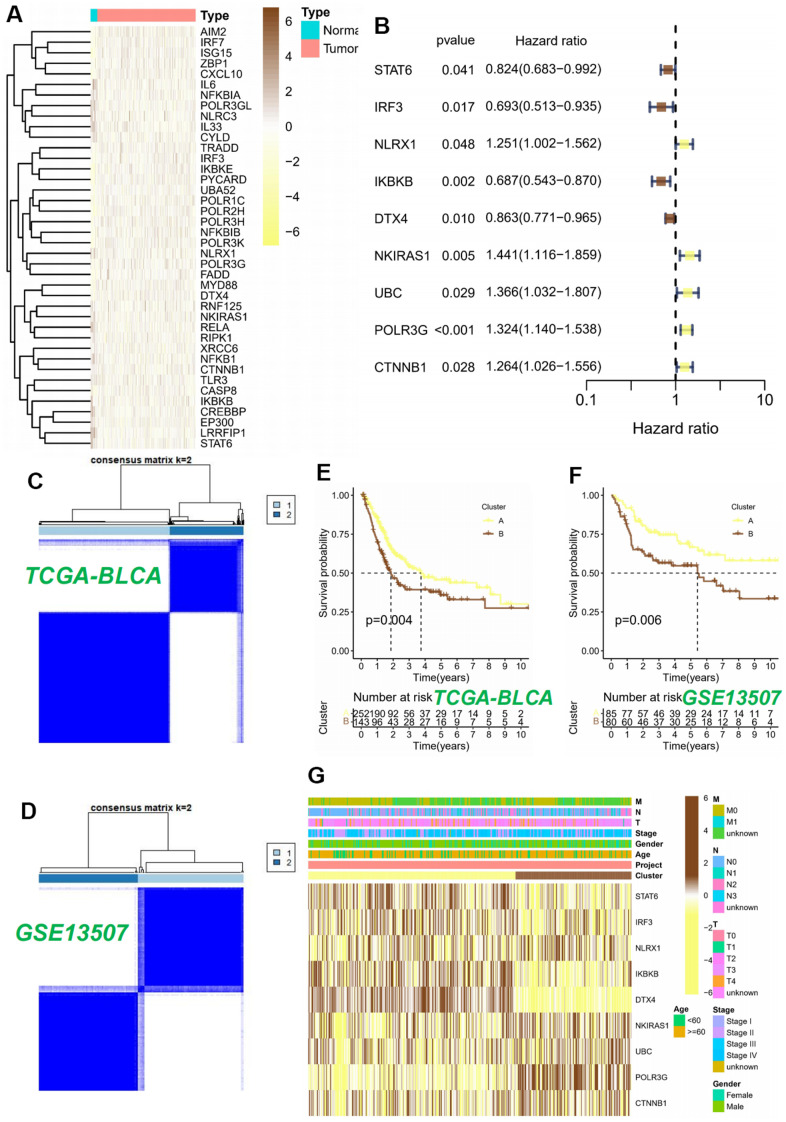
**Consensus clustering analysis of 9 CRGs.** (**A**) The heatmap of 40 differentially expressed CRGs in BLCA tissues. (**B**) Univariable Cox regression analysis of CRGs for the identification of genes associated with prognostic outcomes. (**C**, **D**) Heatmap of the consensus matrices for k=2. Kaplan-Meier curves based on two clusters in the TCGA-BLCA cohort (**E**) and GSE13507 cohort (**F**). (**G**) Heatmap and the clinical parameters of the two clusters.

The procedural framework of this study was elucidated in Figure 1. Initially, we discerned the distinct expression patterns of CRGs within the TCGA cohort. The research findings revealed the existence of 46 differential CRGs. Amongst these, the heatmap illustrated the significant differences top 40 CRGs (Figure 2A). Subsequently, among the aforementioned 46 CRGs, a univariate Cox regression analysis was employed to ascertain nine CRGs that exhibited independent association with the OS of BLCA patients (*P* < 0.05, Figure 2B). Furthermore, utilizing the aforementioned expression profiles composed of nine CRGs in the TCGA-BLCA cohort, we classified all tumor samples into two distinct subtypes (Figure 2C). The analysis of survival indicated a considerable disparity in overall survival between subtype A and subtype B, with subtype B exhibiting a significantly inferior survival rate (*P* < 0.05, Figure 2E). To further ascertain the robustness of this classification scheme, we conducted validation on the GSE13507 cohort. The results indicated that the expression profiles derived from the aforementioned CRGs classified the GSE13507 cohort into two subtypes (Figure 2D). Moreover, the subtype B exhibited an unfavorable prognosis as well (*P* < 0.05, Figure 2F).

Ultimately, we successfully established a substantial association between the two subtypes and CRGs, as well as relevant clinical data within the TCGA-BLCA cohort. The study’s findings unveiled a plausible elucidation for the disparate modifications observed in the cGAS-STING pathway. This disparity could be attributed to variations in the expression of CRGs. Specifically, subtype B exhibited increased expression levels of NKIRAS1, UBC, POLR3G, and CTNNB1, while subtype A showed elevated expression levels of STAT6, IRF3, NLRX1, IKBKB, and DTX4 (Figure 2G).

### Building a predictive signature

**Figure 3:**
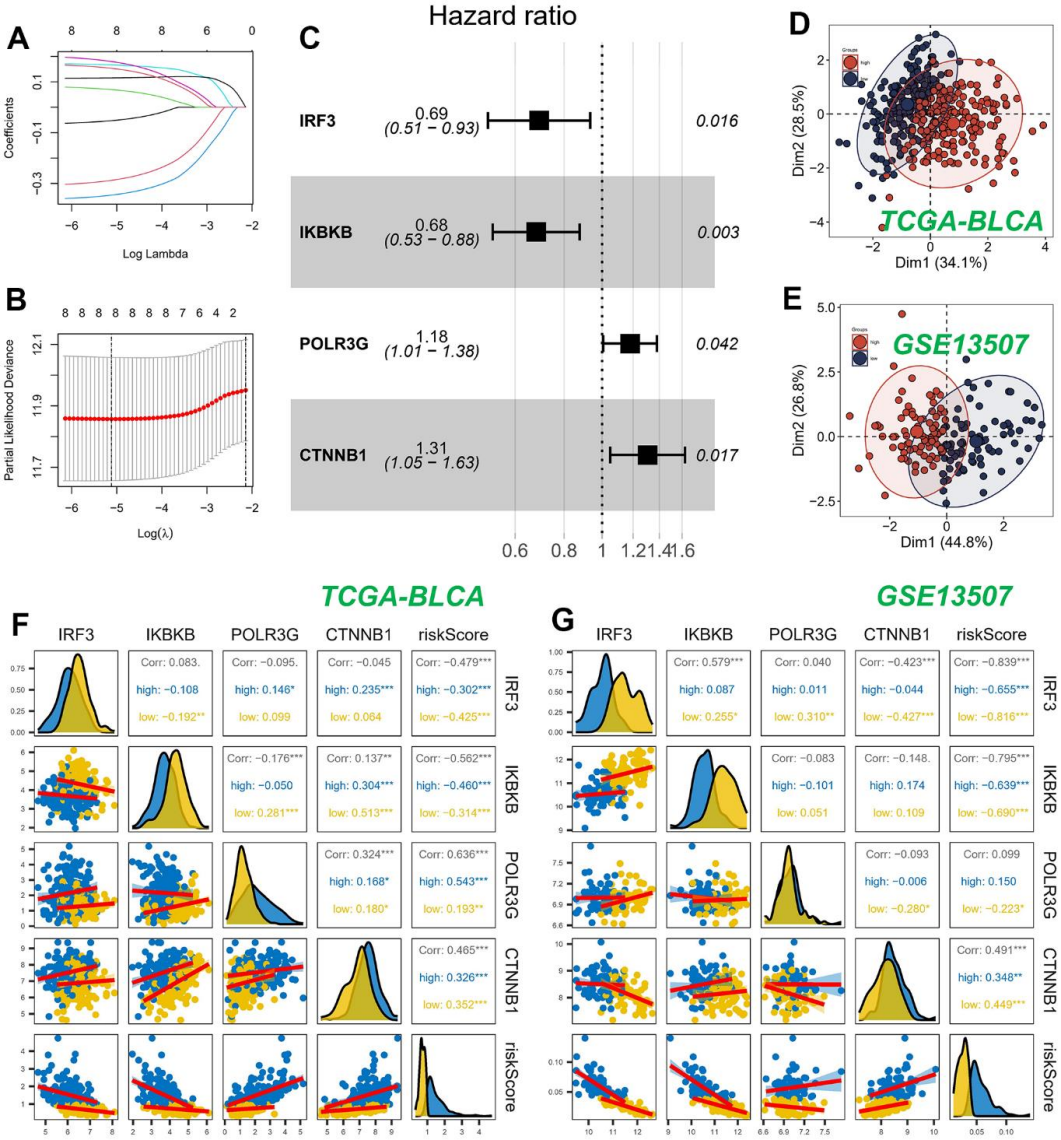
**Construction and validation of CRGs prognostic model.** (**A**, **B**) Nine genes were selected for multivariate regression analysis using Lasso regression. (**C**) A forest plot was utilized to depict the results of a multivariate Cox regression analysis of CRGs. (**D**, **E**) PCA analysis in TCGA cohort. (**F**, **G**) Analysis of the correlation between genes of the model and risk score.

We constructed a prognostic model employing nine CRGs, identified through univariate Cox regression analysis on the TCGA training dataset, which exhibited statistically significant correlation with OS. The study employed LASSO Cox regression and multivariate Cox regression analysis to ascertain a definitive set of four genes (Figures 3A, 3B). The risk score was determined by taking into account the coefficients and expression patterns of four essential genes integrated within the model (Figure 3C). Risk score = (-0.3699 × IRF3 expression) + (-0.3873 × IKBKB expression) + (0.1642 × POLR3G expression) + (0.2673 × CTNNB1 expression). Principal component analysis (PCA) was utilized to effectively segregate the TCGA and GSE13507 datasets into two distinct groups based on the risk score (Figures 3D, 3E). The assessment of gene expression levels indicated a prevailing trend of positive correlations among the model genes. Notably, IRF3 and IKBKB exhibited the highest positive correlations with POLR3G and CTNNB1, respectively. Our observations revealed a strong association between the genes and the model risk score. Specifically, IRF3 and IKBKB displayed substantial negative correlations, whereas POLR3G and CTNNB1 exhibited significant positive correlations (Figures 3F, 3G).

**Figure 4:**
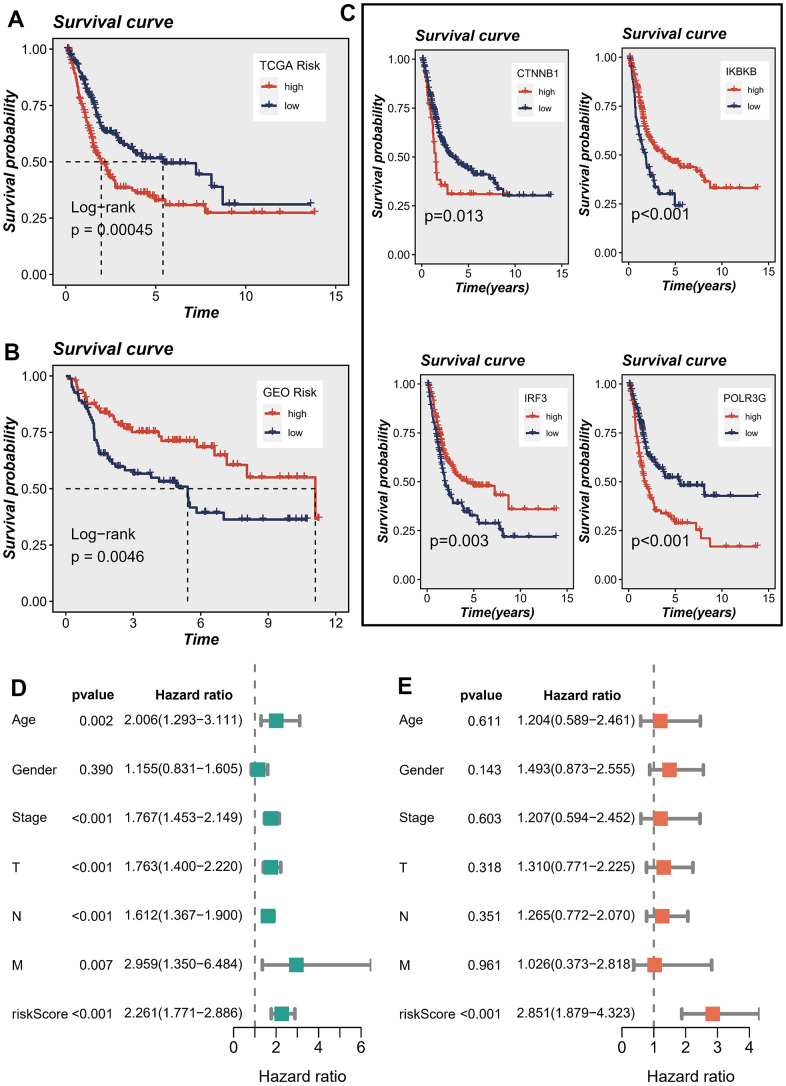
**Correlations between model genes and their impact on survival.** (**A**, **B**) Prognostic analysis of signatures in the TCGA and GSE13507 datasets utilizing Kaplan-Meier method. (**C**) The association between model genes and survival outcomes. (**D**, **E**) The forest plot presents the outcomes of both univariate and multivariate Cox regression analyses, examining the correlation between OS and clinicopathological factors, including the risk score, in patients diagnosed with BLCA.

The patient population was stratified into high-risk and low-risk groups based on the median risk score, serving as criteria for classification. Survival analysis using the Kaplan-Meier method revealed that, within the TCGA cohort, patients classified as high-risk exhibited significantly inferior OS outcomes when compared to their low-risk counterparts (Figure 4A). In contrast, within the GEO cohort, patients classified as high-risk exhibited notably prolonged OS compared to those categorized as low-risk (Figure 4B). Figure 4C illustrated the influence of individual genes on survival. The findings suggested that the IRF3 and IKBKB genes exhibited a protective effect, implying that downregulating the expression of these genes could potentially improve patients’ OS. On the contrary, POLR3G and CTNNB1 were identified as genes associated with high risk, and their upregulated expression was linked to a decrease in patient survival.

In order to assess the potential of the model risk score as a standalone prognostic indicator for survival in BLCA, we conducted a comprehensive analysis. This study encompassed the assessment of the hazard ratio (R) for OS by considering the risk score alongside various pertinent clinical variables, including age, gender, pathology T stage, pathology N stage, pathology M stage, and clinical stage. The univariate Cox regression analysis demonstrated statistically significant associations between OS and several factors, encompassing the risk score, age, T stage, N stage, M stage, and clinical stage (Figure 4D). Upon inclusion of these factors in the multivariate Cox regression analysis, the results revealed that only the risk score maintained a robust association with the prognosis. This finding provides substantial evidence supporting the use of the risk score as an independent factor in predicting prognosis among individuals diagnosed with BLCA (Figure 4E). The aforementioned evidence established a significant association between the proposed risk model and survival outcomes in patients diagnosed with BLCA. Additionally, we conducted a comparative analysis of the prognostic signatures from other studies to further evaluate the accuracy of our own signature. The findings yielded promising results. Specifically, when predicting the TCGA-BLCA cohort, our risk signature exhibited superior predictive value compared to the signatures proposed by Cao et al., Song et al., and Wu et al. [18–20] (*P* < 0.05), as illustrated in Supplementary Figure 1.

### Nomogram construction

**Figure 5:**
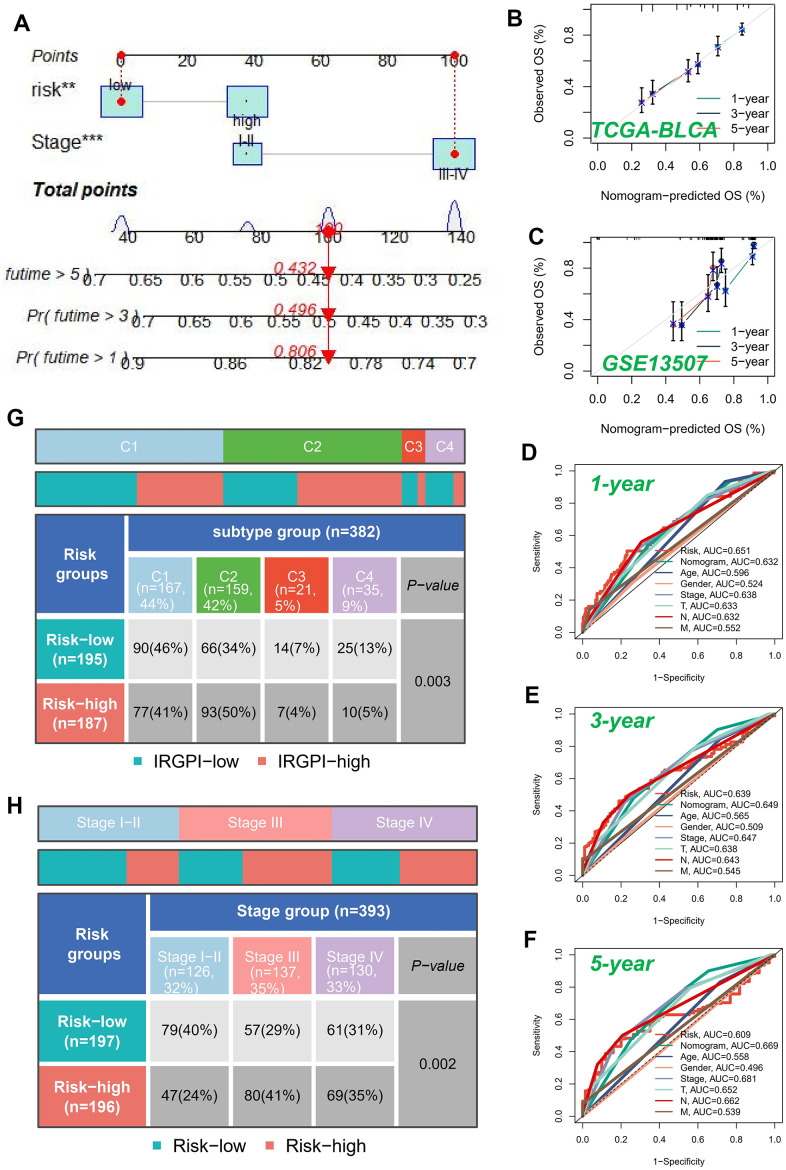
**Building a more accurate nomogram.** (**A**) Nomogram was developed by integrating clinical characteristics with risk stratification. The calibration curves of the nomogram for predicting the probabilities of 1-year, 3-year, and 5-year outcomes were assessed in two independent cohorts: TCGA-BLCA cohort (**B**) and GSE13507 cohort (**C**). (**D**–**F**) ROC curves for 1, 3, and 5 years showed AUC values for various clinical factors, risk scores, and nomogram scores. (**G**) Correlation between high-risk and low-risk groups and four immune subtypes. (**H**) Relationship between tumor stages and diverse risk categories.

A nomogram was devised to assess the risk of TCGA patients by integrating clinical data and risk score. Since the P-value of most clinical parameters was greater than 0.05 in the multivariate regression analysis, we only selected the tumor stage that had the greatest impact on patient survival under clinical experience in the construction of the nomogram. Figure 5A visually represents the comprehensive stage and risk categorization of the patients. This nomogram could greatly enhance the accuracy in assessing patient risk and provide valuable guidance for subsequent therapeutic approaches. To augment the assessment of the accuracy of this nomogram, a prognostic ROC analysis was performed. The findings revealed a remarkable superiority of the outcomes compared to alternative clinical models and risk score. Based on the findings, the AUC values at 1, 3, and 5 years were determined as 0.632, 0.649, and 0.669, respectively, as illustrated in Figure 5D–5F. Moreover, in order to assess the validity of the newly devised nomogram in prognosticating the outcomes of individuals with BLCA over one, three, and five years, calibration curves were produced (Figure 5B, 5C).

The subsequent investigation aimed to examine the association between model categorization and immune subtypes, as depicted in Figure 5G. The immune subtype C2 exhibited a predominance within the high-risk group. Previous studies have demonstrated that the immune subtype C2 cluster displayed the most promising prognosis, while the C4 cluster was predominantly observed in the low-risk classification, consistent with earlier investigations. Moreover, we conducted an exploratory analysis to assess the association between the well-established risk model and the clinical stage of individuals diagnosed with BLCA (Figure 5H). Our analysis revealed a notable trend wherein the proportion of high-risk patients increased as the pathological stage progressed. This finding underscored the robustness and credibility of our developed risk model.

### Analysis of enrichment

**Figure 6:**
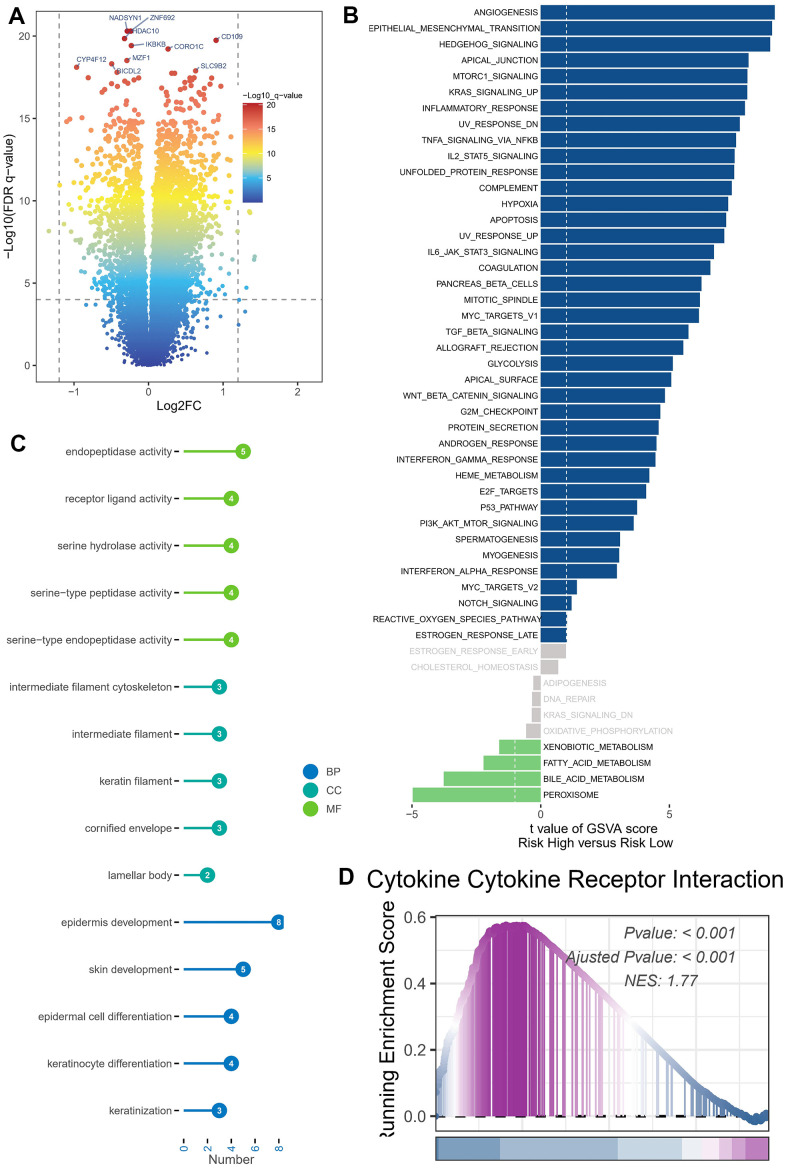
**Enrichment analysis and functional annotation.** (**A**) Differentially expressed genes between high- and low-risk groups (FDR <0.05, log2 (FC) > 1). (**B**) The GSVA analysis demonstrates the enrichment of hallmark gene sets within various risk groups. (**C**) Bar graphs are commonly utilized to show GO enrichment analysis. (**D**) The GSEA enrichment analysis method revealed notable variations in pathways between groups classified as high risk and low risk.

DEGs were identified in cohorts characterized by both high and low risks. To enable visual examination, the expression profiles of the DEGs were graphically depicted using a volcano plot, following rigorous criteria (FDR < 0.05 and log2(FC) > 1) as illustrated in Figure 6A. The examination of gene signatures associated with hallmark pathways revealed notable differences between the high-risk and low-risk groups. The high-risk group exhibited significant enrichment in the following top five molecular signatures: angiogenesis, epithelial mesenchymal transition, hedgehog signaling, apical junction, and mTORC1 signaling (Figure 6B).

The DEGs were subjected to GO enrichment analysis (Figure 6C), which unveiled several key biological processes (BP) at play. The aforementioned processes predominantly involve the development of the epidermis and skin, the differentiation of epidermal cells and keratinocytes, as well as the process of keratinization. The cellular component (CC) analysis revealed a predominant representation of several key components, namely the intermediate filament cytoskeleton, intermediate filament structures, keratin filaments, and the cornified envelope. The primary classifications within molecular function (MF) comprised endopeptidase function, receptor ligand interaction, serine hydrolase role, serine-type peptidase function, and serine-type endopeptidase activity. GSEA was conducted on high- and low-risk BLCA groups (Figure 6D). The GSEA enrichment analysis revealed that the top-scoring NES corresponded to the cellular receptor interplay pathway involving cytokines. Specifically, the pathway involving cellular receptor interplay in high-risk BLCA patients was significantly activated (NES = 1.77, *P* < 0.001).

### Assessment of immune microenvironment

**Figure 7:**
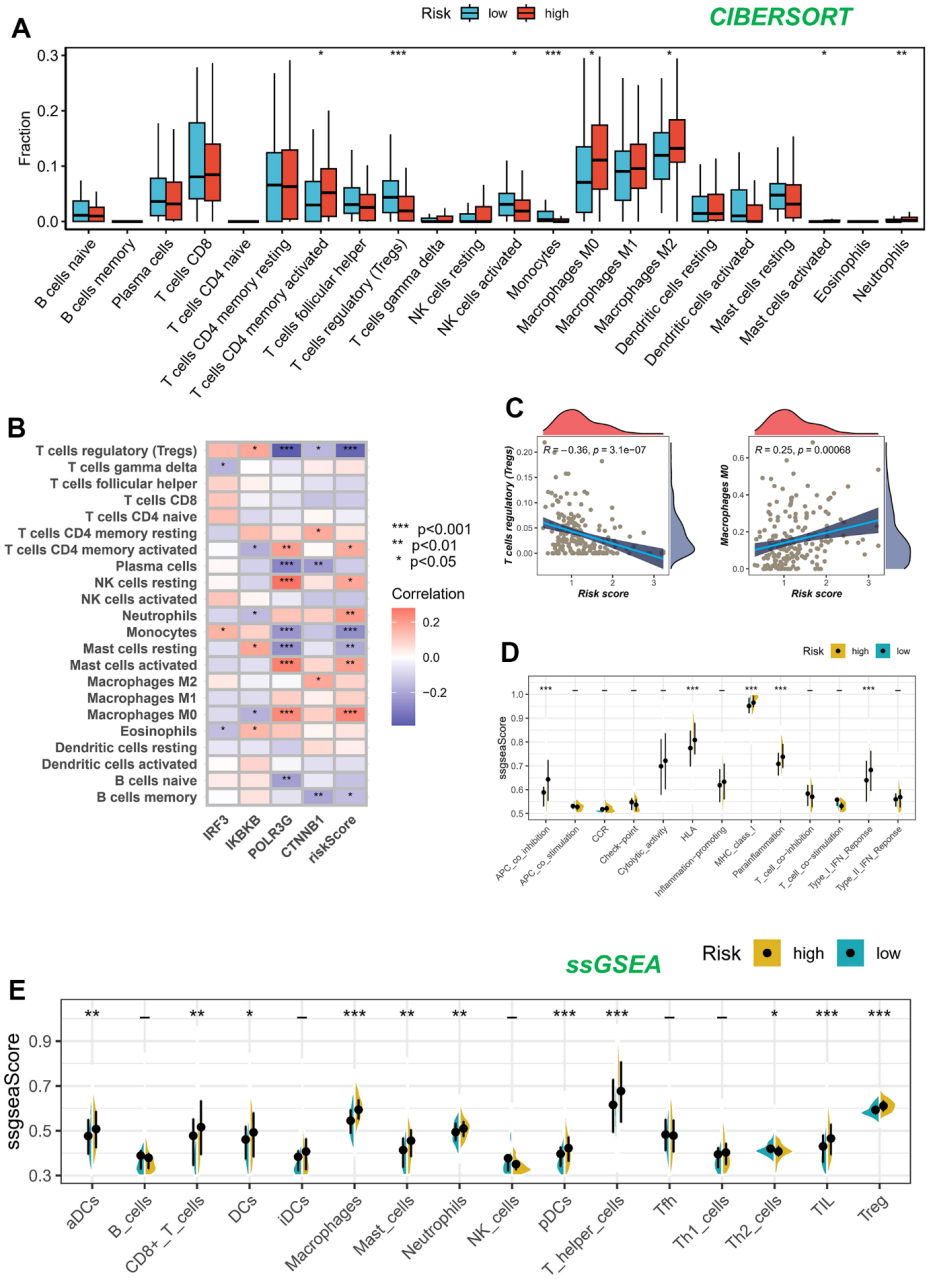
**Analysis of immune microenvironment.** (**A**) Assessing disparities in immune cell content among high and low-risk cohorts. (**B**, **C**) Association between immune cell content and model genes and risk scores. (**D**, **E**) The ssGSEA methodology was employed to assess the disparities in immune cells and immune-associated functions among groups categorized as high risk and low risk.

We utilized the CIBERSORT technique to analyze samples of BLCA, aiming to determine the composition of immune cells in both high-risk and low-risk groups, while investigating relationships between model genes, and risk score. As depicted in Figure 7A, the composition of regulatory T (Treg) cells, activated NK cells, and monocytes was comparatively diminished within the high-risk cohort (*P* < 0.05), whereas memory-activated CD4+T cells, M2 macrophages, M0 macrophages, activated mast cells, and neutrophils exhibited greater prominence in the high-risk population (*P* < 0.05). We also assessed the relationship between the five genes in the proposed model and the abundance of immune cells. We observed that most immune cells were significantly correlated with the five genes (Figure 7B). Figure 7C illustrated the inverse correlation between the risk score and the relative abundance of Treg cells, while displaying a positive correlation with M0 macrophages.

Figure 7D, 7E present the results of the ssGSEA regarding the enrichment of immune cells and immune-related functions. The distribution of immune cells emphasized the distinctive tumor immune microenvironments (TIME) within the two BLCA risk categories. The majority of immune cell types displayed elevated abundance within the low-risk cohort. The low-risk cohort presumably harbored a superior aptitude for eliciting the adaptive immune response owing to their heightened human leukocyte antigen (HLA) functioning, conceivably elucidating the augmented OS observed within this particular group.

### Immunotherapy cohort analysis

The advent of immunotherapy, exemplified by the utilization of PD-1/PD-L1 checkpoint inhibitors, marks a significant breakthrough in the management of neoplastic conditions. In light of the considerable therapeutic prospects offered by immunotherapy, particularly with regard to PD-1 and PD-L1 immune checkpoint inhibitors, in various types of malignancies, including BLCA, we undertook a thorough assessment to determine the prognostic implications of the risk score within the IMvigor210 and GSE78220 cohorts.

**Figure 8:**
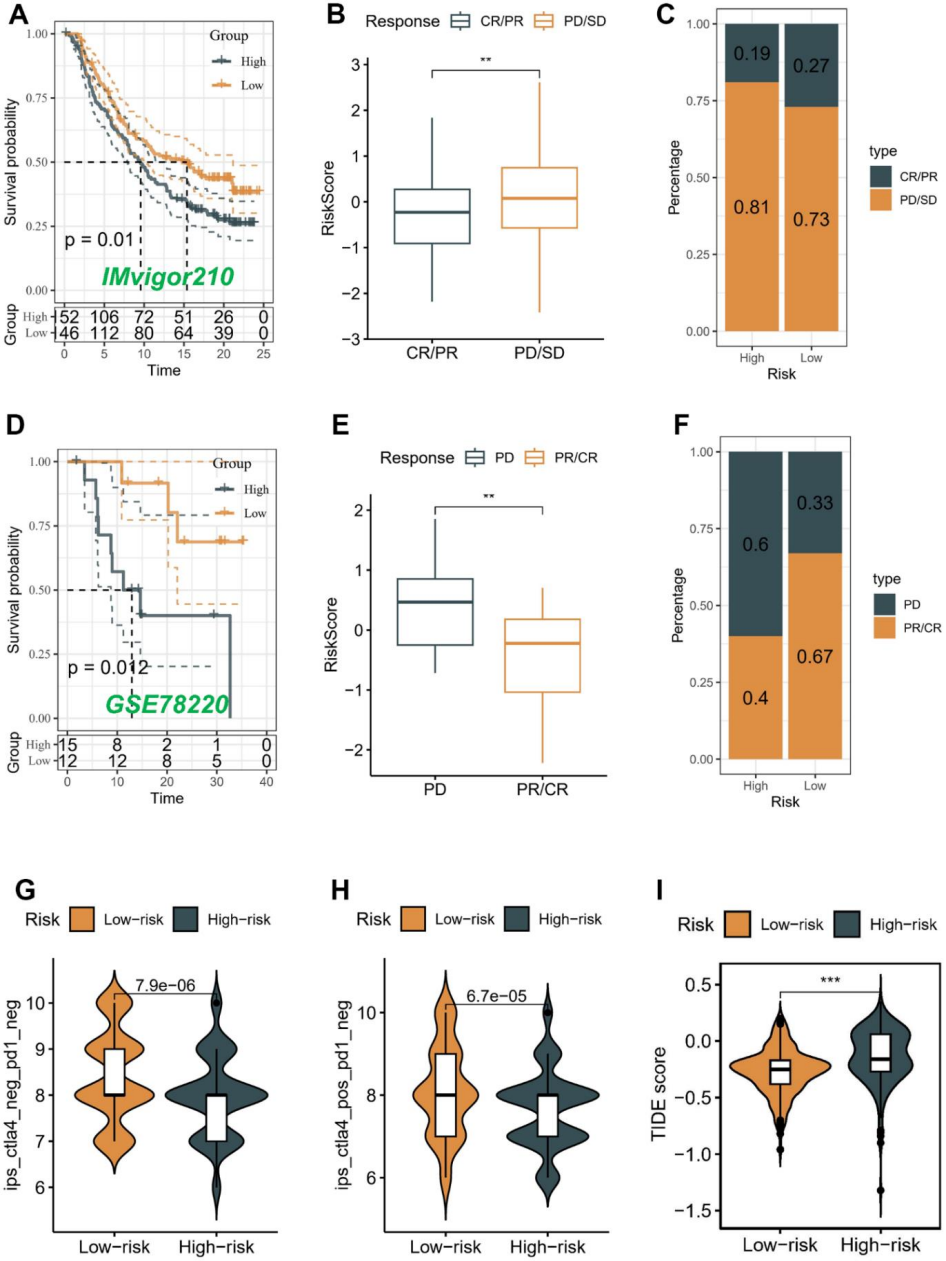
**Correlation of the risk score with immunotherapy response in two cohorts.** Survival analyses (**A**), Distribution of risk score in different immunotherapy response groups (**B**), and Response to anti-PD-L1 therapy (**C**) between low- and high-risk groups in advanced urothelial cancer cohort (IMvigor210 cohort). Survival analyses (**D**), Distribution of risk score in different immunotherapy response groups (**E**), and Response to anti-PD-1 therapy (**F**) between low- and high-risk groups in melanoma cohort (GSE78220). (**G**–**I**) Differences in IPS and TIDE among individuals categorized as high- and low-risk.

We conducted an evaluation of the association between the risk score and the response to immune therapy in the IMvigor210 cohort. This cohort comprised patients diagnosed with advanced urothelial carcinoma who underwent treatment with PD-L1 blockade. Patients classified as low-risk exhibited substantial survival advantages in contrast to individuals categorized as high-risk (Figure 8A). The risk score of patients belonging to the progressive disease (PD)/stable disease (SD) group demonstrated a noteworthy increase when compared to the partial response (PR)/complete response (CR) group (Figure 8B). It is noteworthy to highlight that the incidence of PR/CR within the high-risk group was considerably lower compared to the low-risk group. On the contrary, there was a contrasting pattern observed in the proportion of patients with PD/SD, indicating that the implementation of a risk score holds promise in elucidating the therapeutic efficacy of patients undergoing ICB therapy (Figure 8C).

Conversely, a comparable analysis was performed within the GSE78220 cohort, which consisted of patients diagnosed with melanoma and undergoing PD-1 blockade therapy. In contrast to patients categorized in the high-risk group, those belonging to the low-risk group demonstrated notable benefits in terms of survival outcomes (Figure 8D). The risk score in the PD group demonstrated a considerable elevation when compared to the PR/CR group (*P* < 0.05) (Figure 8E). The prevalence of the low-risk score was notably higher (67%) among patients in the PR/CR group, while the high-risk score was a prominent subtype (60%) within the PD group. These findings suggest that the risk score has potential as a promising predictive marker for assessing the immunotherapeutic response in patients with BLCA (Figure 8F).

Moreover, the Immune Prognostic Score (IPS) held potential in discerning patients who might have benefited from immunotherapy. The tumor specimens obtained from these individuals were expected to demonstrate positive immunological reactions in relation to PD-1/PD-L1 inhibitors, CTLA4 inhibitors, or a combination of both therapeutic interventions. The administration of CTLA-4 immunotherapy resulted in a substantial increase in IPS scores among patients classified as low-risk, indicating that they would have benefited the most from this type of immunotherapy (*P* < 0.05) (Figure 8G, 8H). However, there was no difference between different risk groups in PD-1 and PD-L1 treatment (data not displayed). We also utilized the TIDE algorithm to predict the response to immunotherapy among distinct risk groups. Our results demonstrated a notable decrease in the TIDE score within the low-risk group (*P* < 0.05) (Figure 8I), implying a higher likelihood of favorable outcomes with immunotherapy for patients classified as low-risk.

### Validation of the expression and alteration of the four genes in BLCA tissues

**Figure 9:**
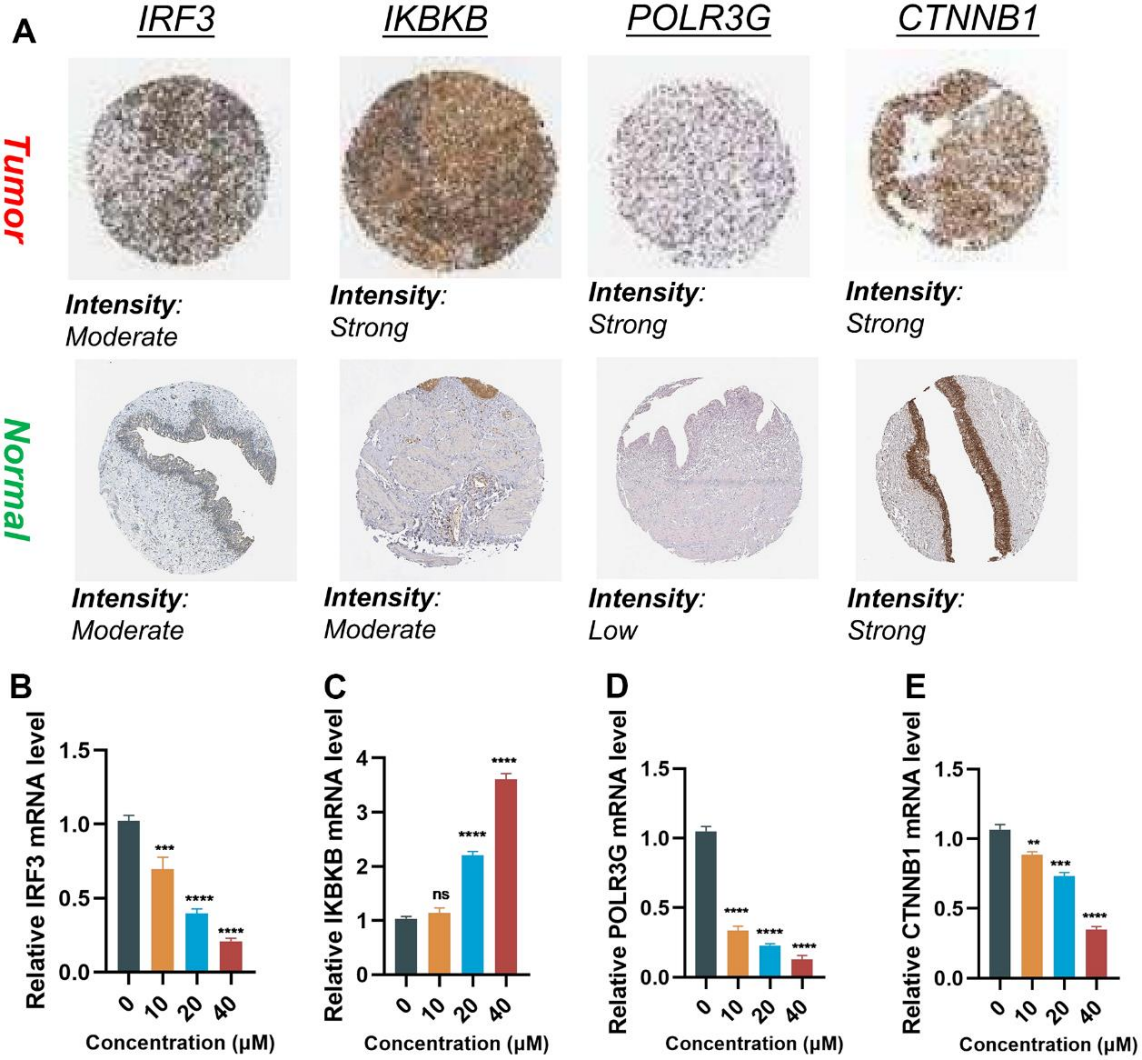
**Validation of the expression of the signature genes in BLCA.** (**A**) Immunohistochemistry of the IRF3, IKBKB, POLR3G, and CTNNB1 in the normal and tumor groups from the HPA database. (**B**–**E**) The expression of Hub gene at different concentrations of AUD-S100.

The protein expressions of the four genes in the BLCA tissues and normal tissues were validated through the utilization of the HPA online database (Figure 9A). The findings demonstrated elevated expression levels of IKBKB and POLR3G in BLCA tissues. IRF3 exhibited a moderate level of expression in both tumor and normal samples. CTNNB1 exhibited high expression levels in both tumor and normal samples.

In order to elucidate whether the expression levels of four genes were influenced by the state of the cGAS-STING pathway, we employed varying concentrations of ADU-S100 (a cGAS-STING pathway agonist) to treat the T24 cell line of BLCA. The RT-qPCR results revealed that alterations in the cGAS-STING pathway status were accompanied by concentration-dependent modifications in the aforementioned quartet of genes. Upon activation of the cGAS-STING pathway, it was observed that the expression of IRF3 and IKBKB was increased (*P* < 0.05), whereas the expression of POLR3G and CTNNB1 was decreased (*P* < 0.05) (Figure 9B–9E).

## DISCUSSION

The cGAS-STING pathway assumes a pivotal function in the advancement and advancement of inflammatory-driven tumors [21]. Prolonged activation of downstream effector programs associated with cGAS-STING leads to persistent inflammation and facilitates tumor progression [22]. Furthermore, the cGAS-STING pathway occupies a central position in DNA sensing, immune cell infiltration, and the regulation of the immune response against tumors [23]. STING-targeted therapies have emerged as a novel category of immunotherapeutic agents for combating tumors. Extensive clinical trials (NCT02675439, NCT03010176, and NCT04144140) have investigated their safety and efficacy. A recent investigation has revealed that the expression of five genes linked to the cGAS-STING pathway, namely IFNB1, IFNA4, IL6, NFKB2, and TRIM25, exhibits potential as a valuable prognostic marker for OS in patients suffering from gastric cancer [24]. Nevertheless, the precise implications of CRGs in BLCA remain uncertain, as there is a paucity of comprehensive understanding in the existing literature. Hence, this investigation represents the inaugural attempt to elucidate the putative role of CRGs in the context of BLCA.

This research investigation discerned two distinct clusters by analyzing the expression matrix of nine genes associated with prognosis. Notably, patients in subtype A exhibited more favorable survival outcomes compared to those in subtype B, as evidenced by the TCGA-BLCA and GSE13507 cohorts. Differential expression patterns were observed in nine prognostic genes between subtypes A and B, indicating distinct regulatory mechanisms of the cGAS-STING pathway within these two clusters. Furthermore, the developed model aimed to discriminate between patients who exhibit a high risk and those who demonstrate a low risk. Specifically, individuals classified as high-risk exhibited a notably poorer survival outcome compared to their counterparts in the low-risk category, as observed within the TCGA-BLCA cohort. In the GSE13507 cohort, it was noted that patients exhibiting a high-risk score exhibited a corresponding elevation in OS rates. The nomogram demonstrated high accuracy in predicting the survival probability of individual patients with BLCA at 1, 3, and 5 years. Additionally, the reliability of the nomogram was confirmed by the calibration curves. Significantly, the risk score exhibits the ability to discern distinct immune subtypes. Moreover, an escalation in the pathological stage corresponds to a notable augmentation in the proportion of patients categorized as high-risk. The results of this investigation strongly endorsed the concept that the model risk score exhibits great potential as a prospective prognostic marker, presenting a valuable approach for optimizing clinical outcomes in individuals afflicted with BLCA.

The proposed model consists of four genes associated with prognosis, namely IRF3, IKBKB, POLR3G, and CTNNB1. The interferon regulatory factors (IRF) comprise a transcription factor family, encompassing nine gene members from IRF1 to IRF9. IRF has been demonstrated to play a pivotal role in immune response and tumorigenesis [25]. In the context of cGAS-STING activation, IRF3 is phosphorylated and translocates to the nucleus, where it activates the transcription of type I interferons and pro-inflammatory cytokines. The cGAS-STING pathway, triggered by DNA present in the cytoplasm, facilitates the activation of two key transcription factors, namely interferon regulatory factor 3 (IRF3) and nuclear factor-κB (NF-κB). This activation subsequently results in the elevation of inflammatory factors and interferons (IFNs) [26]. IKKβ/IKBKB, referred to as IκB kinase beta or IKK2, acquired its nomenclature due to its role in the phosphorylation of IκB molecules, which function as inhibitors of NF-κB transcription factors [27]. Emerging research has elucidated the potential impact of IKBKB activity on tumor development, highlighting its ability to exert negative regulatory effects on key proteins involved in cell cycle regulation and tumor suppression, including members of the p53 family, p16, and TSC1 [28]. According to existing reports, the inhibition of POLR3G has demonstrated a specific halt in proliferation and induction of cell death in prostate cancer cells [29]. Additionally, a correlation has been observed between the overexpression of POLR3G and unfavorable prognostic outcomes in transitional cell carcinoma [30]. The specific role of POLR3G in the context of cGAS-STING activation and bladder cancer is not well-established, and further research is needed to elucidate its significance. β-catenin (CTNNB1) serves as a pivotal regulatory factor within the Wnt signaling pathway, playing a critical role in maintaining tissue homeostasis and governing essential cellular processes such as proliferation, differentiation, and functional regulation. The role of the mutated CTNNB1 in activating the oncogenic Wnt/β-catenin pathway has been documented as crucial in the development of multiple malignancies [31–33]. CTNNB1 has been reported to negatively regulate the immune response by inhibiting the production of type I interferons. The activation of cGAS-STING can lead to the degradation of β-catenin, allowing for enhanced immune responses against bladder cancer. The present study demonstrated that four prognostic-associated genes identified through screening possess functional implications in tumorigenesis and disease progression. The aforementioned findings have provided valuable insights into the importance of these genes in the prognostication of patient survival and the assessment of immunotherapeutic efficacy.

The unique characteristics of cGAS-STING genes in BLCA are hypothesized to be associated with the particular tumor microenvironment. BLCA has a high level of immune invasion [34, 35]. We conducted an in-depth analysis of the variations in infiltration ratios among 22 distinct immune cell populations within both the high-risk and low-risk groups. The high-risk group exhibited a comparatively low infiltration proportion of Treg cells, activated NK cells, and monocytes. In contrast, the high-risk group exhibited a higher prevalence of memory-activated CD4+T cells, M2 macrophages, M0 macrophages, activated mast cells, and neutrophils. Furthermore, our findings unveiled a positive correlation between the risk score and M0 macrophages, neutrophils, NK cells, and memory-activated CD4+T cells, while Treg cells exhibited a negative association with the risk score. Moreover, we corroborated our findings by incorporating two independent external immunotherapy cohorts and employing the IPS to verify that low-risk patients were more likely to benefit from immunotherapy.

In order to ascertain whether the expression of four genes was influenced by the state of the cGAS-STING pathway, we employed varying concentrations of ADU-S100 (a cGAS-STING pathway agonist) to treat the T24 bladder cancer cell line, thereby inducing alterations in their expression levels. The RT-qPCR results demonstrated that alterations in the cGAS-STING pathway status were accompanied by concentration-dependent changes in the aforementioned four genes. To summarize, the activation of the cGAS-STING pathway resulted in the upregulation of IRF3 and IKBKB, while leading to the downregulation of POLR3G and CTNNB1.

While previous articles have investigated the role of the cGAS-STING pathway in BLCA, there are several aspects that make our work novel and distinct: 1-Prognostic Risk Model: We have developed a prognostic risk model using four key genes (IRF3, IKBKB, POLR3G, and CTNNB1) associated with the cGAS-STING pathway. This risk model enables the prediction of OS in BLCA patients. To the best of our knowledge, no previous study has specifically utilized these genes in a prognostic risk model for BLCA. 2-Integration of Clinical Information: In addition to the gene-based risk model, we have established an integrated nomogram that combines risk scoring with clinical information. This approach enhances the clinical practicality of our findings by allowing for a more comprehensive assessment of patient prognosis. 3-Tumor Microenvironment Differentiation: Our study demonstrates that the risk score derived from the CRG model can differentiate tumor microenvironments among different risk groups. This finding provides valuable insights into the underlying mechanisms of BLCA progression and potential targets for personalized immunotherapy. 4-Validation and External Data: We have validated our risk model using an external validation set obtained from the GEO database. This step further strengthens the reliability and generalizability of our findings.

## CONCLUSIONS

In summary, our study has successfully devised a robust prognostic model that demonstrates effectiveness in predicting both the prognosis and responsiveness to immunotherapy among patients with BLCA. Our study findings suggest that IRF3, IKBKB, POLR3G, and CTNNB1 hold promise as viable therapeutic targets for individuals diagnosed with BLCA.

## Supplementary Material

Supplementary Figure 1
