# Supplementary material for: Molecular map of cGAS-STING pathway-related genes in bladder cancer: the perspective toward immune microenvironment and prognosis
Source: Aging (Albany NY). 2024 Jan 17;16(2):1516–35. doi: 10.18632/aging.205442 (PMC10866408; doi:10.18632/aging.205442)
Supplement: Supplementary Figure 1 [file aging-16-205442-s001.pdf]

SUPPLEMENTARY FIGURE

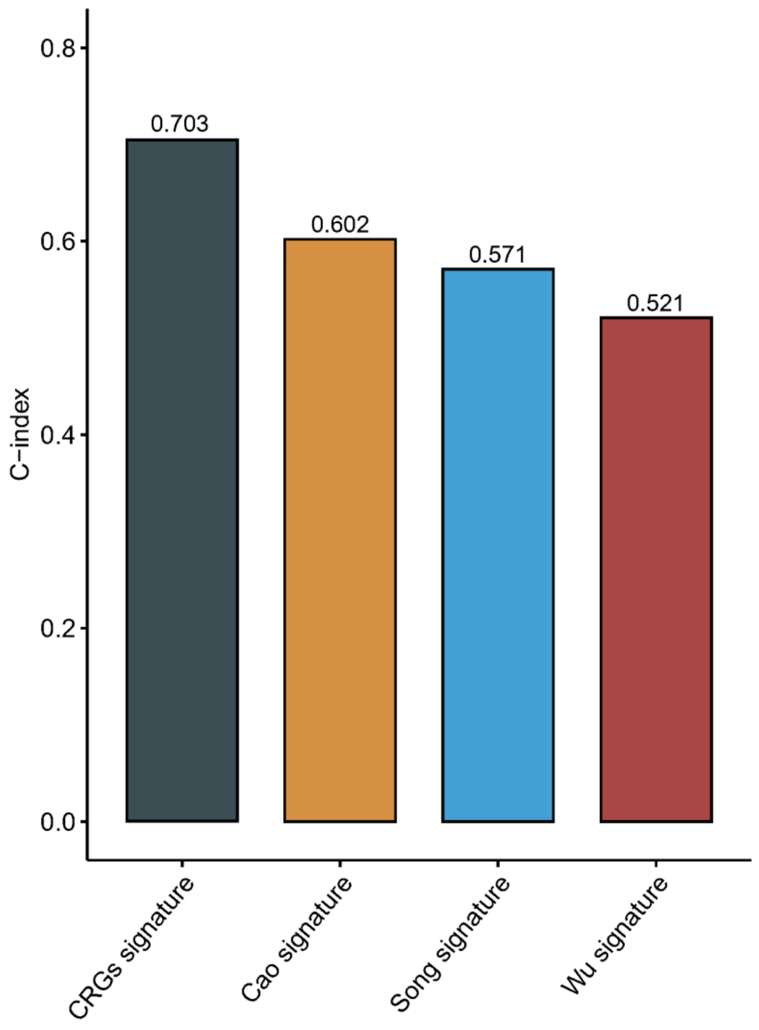

Supplementary Figure 1. Comparison of C-index of different prognostic signatures.
